# Standardization Initiatives in the (eco)toxicogenomics Domain: A Review

**DOI:** 10.1002/cfg.447

**Published:** 2004-12

**Authors:** Susanna Assunta Sansone, Norman Morrison, Philippe Rocca-Serra, Jennifer Fostel

**Affiliations:** 1 EMBL-EBI The European Bioinformatics Institute Wellcome Trust Genome Campus Cambridge CB10 1SD United Kingdom; 2 The University of Manchester Kilburn Building School of Computer Science Oxford Road Manchester M13 9PL United Kingdom; 3 National Institute of Environmental Health Sciences National Center for Toxicogenomics. Research Triangle Park NC 27709 USA

## Abstract

The purpose of this document is to provide readers with a resource of different ongoing standardization efforts within the ‘omics’ (genomic, proteomics, metabolomics) and related communities, with particular focus on toxicological and environmental applications. The review includes initiatives within the research community as well as in the regulatory arena. It addresses data management issues (format and reporting structures for the exchange of information) and database interoperability, highlighting key objectives, target audience and participants. A considerable amount of work still needs to be done and, ideally, collaboration should be optimized and duplication and incompatibility should be avoided where possible. The consequence of failing to deliver data standards is an escalation in the burden and cost of data management tasks.


**Comparative and Functional Genomics**
*Comp Funct Genom* 2004; **5**: 633–641.
Published online in Wiley InterScience (www.interscience.wiley.com). **DOI:** 10.1002/cfg.447

Conference Paper

### Standardization initiatives in the (eco)toxicogenomics domain: a review

Susanna Assunta Sansone[1]*, Norman Morrison[2], Philippe Rocca-Serra[1] and Jennifer Fostel[3]
[1] *EMBL-EBI, The European Bioinformatics Institute, Cambridge CB10 1SD, UK*
[2] *The University of Manchester, Kilburn Building, School of Computer Science, Oxford Road, Manchester M13 9PL, UK*
[3] *National Institute of Environmental Health Sciences, National Center for Toxicogenomics. Research Triangle Park, NC 27709, USA*

*Correspondence to: Susanna Assunta Sansone, EMBL-EBI, The European Bioinformatics Institute, Wellcome Trust Genome Campus, Cambridge CB10 1SD, UK. E-mail: sansone@ebi.ac.uk*

Revised: 24 November 2004
Accepted: 26 November 2004

### Abstract

**The purpose of this document is to provide readers with a resource of different ongoing standardization efforts within the 'omics' (genomic, proteomics, metabolomics) and related communities, with particular focus on toxicological and environmental applications. The review includes initiatives within the research community as well as in the regulatory arena. It addresses data management issues (format and reporting structures for the exchange of information) and database interoperability, highlighting key objectives, target audience and participants. A considerable amount of work still needs to be done and, ideally, collaboration should be optimized and duplication and incompatibility should be avoided where possible. The consequence of failing to deliver data standards is an escalation in the burden and cost of data management tasks. Copyright © 2005 John Wiley & Sons, Ltd.**

**Keywords:** toxicogenomics; ecotoxicogenomics; toxicology; environment; functional genomics; standards; database

### Introduction

Molecular-based approaches, such as transcriptomics, proteomics, metabolomics and metabonomics, are being used to study the impact of chemicals on human and wildlife populations. These high-throughput (eco)toxicogenomics investigations are information-intensive and, by producing massive amounts of data, have placed the informatics challenge under the spotlight. The need to provide easy access to integrated data in a structured standard format is clearly significant. Several efforts are already under way to promote standardization, tackle data management issues and develop databases to facilitate data exchange. We have seen the value of these collaborative efforts already. The Microarray Gene Expression Data (MGED; **http://www.mged.org**) Society has been successful in developing the MIAME standard and related ontology and object models for microarray data (reviewed in Quackenbush 2004). The Reporting Structure for Biological Investigations (RSBI; **http://www.mged.org/Workgroups/rsbi**) is a new working group formed under the MGED Society umbrella, planning to act as a 'single point of focus' for Toxicogenomics, Environmental Genomics and Nutrigenomics communities working towards an international and compatible informatics platform for data exchange. Discipline-specific initiatives are regarded as important because they target 'real world' data capture requirements for the particular omics technologies being used. A consequence of this, however, is that, by remaining within each given discipline, the standardization effort fragments, resulting in duplication and the development of different terminology and data models, thereby limiting the potential for data exchange. One of the objectives of the RSBI working group is to ensure that these initiatives are coordinated, so that synergy and cross-discipline communication can be maximized, and duplicated effort can be minimized.

Copyright © 2005 John Wiley & Sons, Ltd.

To capitalize on these efforts, representatives of the RSBI working group are also directly participating in certain initiatives and, by fostering interactions, are laying the ground for further collaborations. One forum for such interaction is the Standards and Ontologies for Functional Genomics (SOFG; **http://www.sofg.org**) Conference. We invite comments on the work of the RSBI at **mged-rsbi@lists.sourceforge.net**

### Standardization initiatives

Data standardization is now considered beyond the research application of high-throughput technologies (reviewed in Quackenbush, 2004) and regulatory bodies, such as the US Food and Drug Administration (FDA) and Environmental Protection Agency (EPA), are developing their policy or guidance on genomics data submissions (**http://www.fda.gov/cder/guidance/5900dft.doc; http://www.epa.gov/osa/genomics.htm**). Several organizations and committees are tackling data standardization; however, there is a fundamental difference in both the design and objectives of the efforts around regulatory submission of data vs. the needs of the research community, who need databases and tools for discovery. The former aims to accelerate the review process, facilitate proprietary data submission and optimize data visualization in a way that does not impact the vocabulary used by the individual submitter. The research community needs to ease deposition in public databases and facilitate data mining by the use of common annotation standards and ontologies. There is some overlap between the needs of these communities and some level of interaction. Thus, there is value in assessing the commonality between regulatory, research community and database designers' objectives in the design of data standards. Specifically, a unified approach to describing and reporting the experimental biological metadata that is common to the different 'omics' technologies (transcriptomics, proteomics and metabonomics/metabolomics) or disciplines (e.g. pharmacogenomics, toxicogenomics, environmental genomics) is a goal of the RSBI. Undoubtedly specialized information is needed by certain applications, but a high-level unified model for description of metadata would be able to encompass these applications. Here, metadata, refers to biological information relating to samples and the information about experimental design. Data refers to measured values relating to samples (e.g. toxicological endpoints and gene expression) under given experimental conditions.

This paper is not an exhaustive list of all activity but provides a summary of standardization efforts for toxicological and environmental applications, which address reporting standards (e.g. what should be reported), and management issues (e.g. how reported information should be stored and exchanged, and which ontologies should be used to annotate data and metadata). The various initiatives fall into six broad categories, summarized in Table 1 and explored in detail below.

### 'Omics' technology communities

These are academic grass roots communities that have joined forces with commercial vendors to address content standards and reporting needs for a single high-throughput technology.

### MGED Society

The MGED Society has established standards for microarray data annotation (MIAME; Brazma *et al.*, 2001; Ball *et al.*, 2002) and exchange (MAGE-ML; Spellman *et al.*, 2002) that have facilitated the creation of microarray databases and related supporting software (MAGE-OM; Spellman *et al.*, 2002). The response from the scientific community to these community standards has been extremely positive (Editorial, 2002). Most of the major scientific journals and some funding agencies require publications describing microarray experiments to comply with MIAME, for the data to be submitted to public repositories, such as ArrayExpress (Brazma *et al.*, 2003), GEO (Edgar *et al.*, 2002) and CIBEX (Ikeo *et al.*, 2003). Consequently, the MIAME model has been adopted by other communities (Quackenbush, 2004). MGED is now working with other initiatives, such as HUPO-PSI in the proteomics field and SMRS (see below). There have been several extensions to MIAME: MIAME-Tox, an array-based toxicogenomics standard developed by the ILSI Health and Environmental Sciences Institute (HESI) (**http://hesi.ilsi.org/index.cfm?pubentityid=120**); the National Institute of Environmental

Copyright © 2005 John Wiley & Sons, Ltd. 

**Table 1.** The initiatives divided according to six broad categories

| Category | Description | Acronym | Domain | URL |
|---|---|---|---|---|
| Omics technology communities | Academic grass roots communities that have joined forces with commercial vendors to create technology-driven standards | MGED<br>PSI<br>SMRS | Microarray<br>Proteomics<br>Metabolomics and metabonomics | **http://www.mged.org**<br>**http://psidev.sourceforge.net**<br>**http://www.smrsgroup.org** |
| Measurement and methods validations | Efforts focusing on validation programs and production of standard materials and methods | ECVAM | Array-based toxicogenomics | **http://ecvam.jrc.cec.eu.int** |
| | | ERCC | Microarrays and quantitative RT-PCR | **http://www.cstl.nist.gov/bio-tech/workshops/ERCC2004** |
| | | **MARG** | Microarray | **http://www.abrf.org/index.cfm/group.s how/** |
| | | **ABRF** | | **Microarray.30.htm** |
| | | MFB | | **http://www.mfbprog.org.uk** |
| Regulatory driven discussion fora | Efforts aiming for a broader | CDISC | Clinical data | **http://www.cdisc.org** |
| | understanding and use of omics data, defining data models for data | PGx | Pharmacogenomics data | To be announced |
| | submission to regulators. That preserve the terms and observations used by the submitter | SEND | Animal toxicity data | **http://www.cdisc.org/models/send/v1.5** |
| Domain-driven discussion fora | Efforts aiming to a broader exchange and integration of toxicity and ecological data | DSSTox<br>SEEK | Chemical toxicity data<br>Ecological data | **http://www.epa.gov/nheerl/dsstox/**<br>**http://seek.ecoinformatics.org** |
| World-wide organizations | Efforts producing internationally agreed instruments, decisions and recommendations or acting as facilitator | IPCS<br>NAS<br>OECD<br>BSC IEEE | Toxicogenomics<br>(Eco)toxicogenomics<br>Ecotoxicogenomics<br>Bioscience | **http://www.who.int/ipcs/en/**<br>**http://dels.nas.edu/emerging-issues**<br>**http://www.oecd.org**<br>**http://www.csbcon.org** |
| Infrastructure | Standards-compliant infrastructure, assisting in development of useful and usable standards | ArrayExpress and Tox-MIAMExpress | Array-based data and toxicology endpoints values | **http://www.ebi.ac.uk/array-express**<br>**http://www.ebi.ac.uk/tox-miamexpress** |
| | | CEBS<br>CTD<br>maxd | Toxicogenomics<br>Genes and proteins<br>Array-based data and environmental metadata | **http://cebs.niehs.nih.gov**<br>**http://ctd.mdibl.org**<br>**http://bioinf.man.ac.uk/micro-array/maxd** |
| | | TIS (ArrayTrack) | Toxicogenomics | **http://www.fda.gov/nctr/sci-ence/centers/toxicoinfor-matics/ArrayTrack** |

Health Sciences (NIEHS); the National Center for Toxicogenomics (NCT; **http://www.niehs.nih.gov/nct**); the FDA National Center for Toxicological Research (NCTR; **http://www.fda.gov/nctr**); and the European Bioinformatics Institute (EBI; **http://www.ebi.ac.uk**). MIAME/Env has been developed by the NERC Environmental Genomics Thematic Programme Data Centre (EGTDC; **http://envgen.nox.ac.uk**) to fulfil the diverse needs of those working in the functional genomic of ecosystems, invertebrates and vertebrates which are not covered by the model organism community. However, extending MIAME to meet domain-specific requirements is only a partial solution. As multi-technology investigations become commonplace, these checklists will soon be insufficient. Currently, the above communities are working together with the RSBI group to develop a reporting structure for describing multi-platform technologies investigations. The proposed RSBI Tiered Checklist (RSBI TC; **http://www.mged.org/Workgroups/rsbi**) will be a modular context-dependent structure.

Copyright © 2005 John Wiley & Sons, Ltd. 

### Proteomics Standardization Initiative (PSI)

The HUPO (Human Proteome Organization; (**http://www.hupo.org**) PSI (**http://psidev.sourceforge.net**) includes the major protein databases, government and industry and is defining standards for data representation in proteomics to facilitate data comparison, exchange and verification. Current focus is on mass spectrometry and protein–protein interaction data. A set of open source standards are being developed along MIAME lines, including a content standard, the Minimum Information About Proteomics Experiments (MIAPE), an XML standard data exchange format (Hermjakob *et al.*, 2004) and an ontology of clearly defined general proteomics terms.

### Standard Metabolic Reporting Structure (SMRS)

SMRS (**http://www.smrsgroup.org**) comprises industry, software developers, governmental representatives and academia, who are investigating the reporting and design of metabonomics and metabolomics studies in plants, microbial systems, environment, *in vivo* and *in vitro* applications, as well as human studies. A set of draft recommendations has been produced as a discussion document. It considers the factors in a metabolic study that could be recorded and standardized, including the origin of a biological sample, the technologies and methods for analysis and the chemometric and statistical approaches. The recommendations also touch on the granularity of information required for different reporting needs, including journal submissions, public databases and regulatory submissions.

### Measurement and methods validations

As high-throughput technologies are used in industry and are considered by regulatory agencies, the methodology itself comes under scrutiny. Agreement on data formats will do little good if experimental protocols are inconsistent. Currently, standardization of microarray experiment procedures is key to the broad acceptance and use of these data. The very variability of microarray data generation, analysis, future validation of the technology and production of standard materials is now the focus of many initiatives.

### MfB (Measurements for Biotechnology) program

MfB (**http://www.mfbprog.org.uk**) is a UK programme that addresses bio-measurements of importance for industry. The 'Comparability of Gene Expression Measurements on Microarrays' is an industry-based consortium led by LGC (**http://www.lgc.co.uk**). The project is designed to determine the accuracy and comparability of gene expression measurements made on different array platforms and also evaluates data analysis methods. A second phase is now looking at the standardization of array-based toxicogenomics and will build up on the analysis framework to develop a panel of quality metrics for validating and standardizing array-based toxicogenomics measurements.

### The Microarray Research Group (MARG) of the Association of Biomolecular Resource Facilities (ABRF)

The MARG (**http://www.abrf.org/index.cfm/group.show/Microarray.30.htm**) is a research-focused consortium of academic laboratories promoting communication and cooperation among core academic and industrial microarray and data analysis services providers. The resulting data is used to help laboratories evaluate their performance and achieve the highest quality results possible from the use of microarray technologies.

### The European Centre for the Validation of Alternative Methods (ECVAM)

The ECVAM (**http://ecvam.jrc.cec.eu.int**) coordinates and funds validation studies of alternative methods that could reduce, refine or replace the use of laboratory animals in regulatory toxicology. Both the new EU Chemical Policy (REACH) (Editorials, 2003a, 2003b) that proposes the re-evaluation of about 30 000 chemicals, and the 7th Amendment to the Cosmetics Directive, which foresees the complete replacement of animal experiments by 2013, call for the development and implementation of alternative methods. ECVAM is working with the US Interagency Coordinating Committee on the Validation of Alternative Methods (ICCVAM; **http://iccvam.niehs.nih.gov/home.htm**) and National Toxicology Program Interagency Center for the Evaluation of Alternative Toxicological Methods (NICEATM; **http://iccvam.ni-**

Copyright © 2005 John Wiley & Sons, Ltd. 

**ehs.nih.gov/home.htm**) to investigate the specific considerations necessary for adequate validation of array-based toxicogenomics-based test methods. At present, recommendations are being prepared which will cover topics such as description of the biological systems, methodological/technical issues, data analysis, and data format and storage.

### External RNA Controls Consortium (ERCC)

ERCC (**http://www.cstl.nist.gov/biotech/workshops/ERCC2004**) originated at a US National Institute of Standards and Technology (NIST; **http://www.nist.gov**) meeting and is composed of representatives from the public, private and academic sectors, addressing experimental control and performance evaluation for gene expression analysis. ERCC is considering the utility of universal (platform-independent) spike-in controls, protocols, and informatics tools intended for use across one- and two-channel microarray and quantitative RT-PCR (QRT-PCR). Outcomes of this work will be published and resulting data submitted to a public database.

### Regulatory-driven fora

To streamline regulatory electronic submissions a number of technical issues need to be addressed. These efforts intend to identify the kind of data that should be included in submissions to regulatory bodies and automate the largely paper-based clinical trials and non-clinical research processes.

### Clinical Data Interchange Standards Consortium (CDISC)

CDISC (**http://www.cdisc.org**) is an open, multidisciplinary, non-profit organization committed to the development of worldwide pharmaceutical industry standards, vendor-neutral, platform-independent data models to support the electronic acquisition, exchange, and the submission and archiving of clinical trials data and metadata.

### Standard for Exchange of Non-clinical Data (SEND)

SEND (**http://www.cdisc.org/models/send/v1.5**) is a consortium formed among the pharmaceutical industry, contract laboratories, software developers and the FDA. The goal of SEND is to develop a common format for the electronic submission of animal toxicity data and study description to a regulatory agency. Once the SEND standard is finalized, it will be merged with CDISC's model to form the Study Data Tabulation Model (SDTM).

### Pharmacogenomics (PGx) Standards Group

The Pharmacogenomics (PGx) Standards Group was formed in November 2003 at a workshop organized by the Drug Information Association (DIA), FDA, Pharmacogenetics Working Group (PWG), Pharmaceutical Research and Manufacturers of America (PhRMA) and Biotechnology Industry Organization (BIO) to review the FDA draft, 'Guidance for Industry — Pharmacogenomic Data Submissions'. The PGx Standards Group encompasses regulatory bodies, pharma, and industry organizations. The goal of this joint project is to help define the requirements for pharmacogenomics submission to the FDA and define data formats and standards. This project focuses on the use of pharmacogenomics and toxicogenomics data to support pharmacological and toxicological conclusions. There is a consensus within this group to use existing standards (e.g. MIAME, MAGE, SEND, CDISC) if available, and to extend them if needed.

### Domain-driven fora

These toxicoinfomatics and ecoinformatics specific initiatives are an example of international coordination for the development and adoption of controlled vocabularies and format for exchanging chemical toxicity, and ecological and environmental data.

### The Distributed Structure-Searchable Toxicity (DSSTox)

DSSTox (**http://www.epa.gov/nheerl/dsstox**) is a network project by the US EPA, providing a community forum for publishing standard format, structure-annotated chemical toxicity data files for open public access. Although a primary focus of this effort is aimed towards inclusion of chemical structures and standardized chemical fields, DSSTox will also promote the use of a controlled vocabulary, i.e. common data field names

Copyright © 2005 John Wiley & Sons, Ltd. 

and entry formats for the same types of toxicity data across databases. It will link to such public toxicity data by incorporating DSSTox Standard Fields and Indices in the custom databases, making common queries possible using a standard DSSTox identifier. DSSTox is collaborating with, or using standards from, several other efforts, including the LeadScope In Silico Tox (LIST) Focus Group, the National Cancer Institute (NCI), NIEHS's National Center for Toxicogenomics and the National Toxicology Program, the National Library of Medicine (NLM) TOXNET, the International Union of Pure and Applied Chemistry (IUPAC), the National Institutes of Standards and Technology (NIST), the ILSI HESI SAR Toxicity Database Project and MGED's MIAME/Tox, as well as numerous vendors and consortia (**http://www.epa.gov/nheerl/dsstox/CoordinatingPublicEfforts.html**).

### The Science Environment for Ecological Knowledge (SEEK)

SEEK (**http://seek.ecoinformatics.org**) is a multidisciplinary initiative designed to create cyberinfrastructure for ecological, environmental and biodiversity research and to educate the ecological community about eco-informatics. SEEK participants are building an integrated data grid (EcoGrid) for accessing a wide variety of ecological and biodiversity data and analytical tools (Kepler; **http://kepler-project.org**). Ecological Metadata Language (EML) is a metadata specification developed in association with SEEK and the Knowledge Network for Biocomplexity (KNB; **http://knb.ecoinformatics.org**) that can by used in a modular and extensible manner to document ecological data.

### World-wide organizations

Global organizations have initiated a dialogue between technological experts, regulators and the principal validation bodies to draw road maps for development, validation and regulatory use of omics-based technologies in chemical assessment. Others are liaising with different life sciences disciplines, offering support, mediation and consultancy to speed up the standards development process.

### Organization for Economic Co-operation and Development (OECD) and the International Program on Chemical Safety (IPCS)

IPCS (**http://www.who.int/ipcs/en/**) is a joint program of three cooperating organizations — the International Labour Organization, the United Nations Environment Network and the World Health Organization — implementing activities related to chemical safety. In collaboration with the Organization for Economic Cooperation and Development (OECD, **http://www.oecd.org**), the IPCS has organized a series of workshops to identify the possible application of methods based on (eco)toxicogenomics in regulatory hazard assessment, to determine the current limitations to the use of (eco)toxicogenomics in regulatory assessment and develop a plan to overcome such limitations, to identify the need for future activities with regard to the use of these methods in test guidelines, new and existing chemicals, pesticides and biocides programs. At present, recommendations are being prepared and will be published. In view of these recommendations, the development of a coordinated international research program on (eco)toxicogenomics will be initiated, aiming to optimize the integration of genomic techniques into (eco)toxicology and their use in ecological and human health risk assessment.

### The National Academy of Sciences (NAS)

The NAS Committee on Emerging Issues and Data on Environmental Contaminants (**http://dels.nas.edu/emergingissues**) is a public forum for communication among government, industry, environmental groups and the academic community about emerging evidence and issues in toxicogenomics, environmental toxicology, risk assessment and exposure assessment. The Committee will develop a framework for how the emerging field of genomics will be incorporated into risk assessment.

### Institute of Electrical and Electronics Engineers (IEEE) Computer Society

The Bioinformatics Standards Committee (BSC; **http://www.csbcon.org**) has a mission to act as a liaison between groups in the bioscience community, developing standards for biological objects

Copyright © 2005 John Wiley & Sons, Ltd. 

in the life sciences disciplines and the IEEE Standards Association. BSC will provide a neutral forum for the global bioinformatics community to work towards common agreements on standards in new areas and integration between established standards.

### Standard(s)-compliant infrastructure

This section provides a short review of public infrastructure currently available for toxicogenomics and environmental genomics data. These efforts are in different stages of development, serving specific needs of their user community and relying on diverse types of funding support. Nevertheless, these are examples of institutions working together, sharing expertise and moving towards an internationally compatible informatics platform for data exchange, interacting closely with standardization initiatives listed here.

### ArrayExpress and Tox-MIAMExpress

ArrayExpress (**http://www.ebi.ac.uk/arrayexpress**) (Brazma *et al.*, 2003) is a MGED standards-compliant, public infrastructure for microarray-based gene expression data at the EBI. The infrastructure has been extended to link biological endpoint values with gene expression data as result of a collaborative undertaking with the ILSI HESI Committee on the Application of Toxicogenomics Data to Mechanism-based Risk Assessment (**http://www.ebi.ac.uk/microarray/Projects/tox-nutri**). Their toxicogenomics datasets (Pennie *et al.*, 2004) have been submitted to ArrayExpress using Tox-MIAMExpress, the online MIAME/Tox-compliant data input tool (Mattes *et al.*, 2004) (**http://www.ebi.ac.uk/tox-miamexpress**). The ILSI HESI Committee research programme has provided the first large array-based toxicogenomics dataset in the public domain annotated according to the MGED standards.

### Chemical Effects in Biological Systems (CEBS) Knowledgebase

CEBS (**http://cebs.niehs.nih.gov**) (Waters *et al.*, 2003) is a public toxicogenomics knowledgebase in year two of its 10 year development at the NIEHS's NCT. CEBS aims to integrate omics datasets in the context of toxicology to advance knowledge discovery about toxicity (Waters *et al.*, 2003; Waters and Fostel, 2004; Mattes *et al.*, 2004). CEBS implements standards developed by the MGED Society and the HUPO PSI in the CEBS SysBio object model (Xirasagar *et al.*, 2004). CEBS is designing an ontological representation of data and terms used by its collaborators, which includes descriptors for different study design types and metadata vocabularies.

### maxd maxd (**http://bioinf.man.ac.uk/microarray/maxd**) is an open-source data warehouse and visualization environment for genomic expression data employed by the NERC EGTDC. The maxd software suite includes two major components. The first, maxdLoad2, is a database schema and data loading and curation application designed to enable biologists to store expression data, annotate it to MIAME and MIAME/Env standards, and export it in MAGE-ML format to ArrayExpress. The second, maxdView, is a modular analysis and visualization environment for interactive exploration of transcriptomics data and associated metadata.

### Toxicoinformatics Integrated System (TIS)

ArrayTrack (**http://www.fda.gov/nctr/science/centers/toxicoinformatics/ArrayTrack**; Tong *et al.* 2003) is an integrated software system for managing, mining and visualizing microarray gene expression data at NCTR-FDA. The system has three integrated components: a MIAME-compliant database storing array-based toxicogenomics data; a set of tools providing data visualization and analysis capability; and a library containing functional information about genes, proteins, pathways and toxicants. ArrayTrack is the first module of TIS, a system to integrate genomic, proteomic and metabonomic data with data from the public repositories, as well as conventional *in vitro* and *in vivo* toxicology data. TIS will serve as a general toxicogenomics repository for diverse data sources, supporting broad data mining and meta-analysis activities, as well as the development of robust and validated predictive toxicology systems.

Copyright © 2005 John Wiley & Sons, Ltd. 

### The Comparative Toxicogenomics Database (CTD)

The CTD (**http://ctd.mdibl.org**) promotes understanding about the effects of environmental chemicals on human health by facilitating cross-species comparative studies of toxicologically important genes and proteins. CTD is now publicly available as a prototype. It provides annotated associations between genes, proteins, sequences, references and chemicals in vertebrates and invertebrates; integrates molecular and toxicology data; implements ontologies; and will describe gene–chemical interactions in diverse organisms. These data provide insight into the genetic basis of variable sensitivity to chemicals and complex interactions between the environment and human health.

### Conclusions

Data produced by (eco)toxicogenomics investigations are growing in volume and complexity at a staggering rate. It is not trivial to define precise data content, presentation and exchange formats. However, there is a growing realization within the (eco)toxicogenomics community that, if we are to realize the opportunities offered by omics-based technologies, we will need to change our approach to data handling and work more collaboratively. The authors, also moderators of the RSBI working group, would like to emphasize the need for community participation in the integration of these standardization initiatives. It is hoped that highlighting these different initiatives will help to assess the commonality and optimize harmonization, thus minimizing duplication and incompatibility and achieving cost-effective results in a timely manner.

### Acknowledgements

The authors would like to thank the following people for their assistance: Alvis Brazma, Ann Richard, Betty Cheng, Carole Foy, Carolyn Mattingly, Chris Taylor, Craig Zwickl, Dawn Field, Helen Parkinson, Henning Hermjakob, Jason Snape, Jessie Kennedy, John Lindon, Michael Waters, Nancy Doerrer, Peter Lord, Raffaella Corvi, Robert Stevens, Syril Petit, Thomas Papoian, Weida Tong and the SOFG organizers. Susanna-Assunta Sansone is supported by the ILSI-HESI Genomics Committee, Norman Morrison by the NERC Environmental Genomics programme, Philippe Rocca-Serra by the European Commission NuGO project and Jennifer Fostel by the NIEHS NCT.

### References

ArrayExpress: **http://www.ebi.ac.uk/arrayexpress**

ArrayTrack: **http://www.fda.gov/nctr/science/centers/toxicoinformatics/ArrayTrack**

Ball CA, Sherlock G, Parkinson H, *et al*. 2002. An open letter to the scientific journals, published in: *Science* **298**(5593): 539; *Bioinformatics* **18**(11): 1409; *Lancet* **360**: 1019.

Brazma A, Parkinson H, Sarkans U, *et al*. 2003. ArrayExpress — a public repository for microarray gene expression data at the EBI. *Nucleic Acids Res* **31**(1): 68–71.

Brazma A, Hingamp P, Quackenbush J, *et al*. 2001. Minimum information about a microarray experiment (MIAME) — toward standards for microarray data. *Nature Genet* **29**(4): 365–371.

BSC; **http://www.csbcon.org**

CDISC; **http://www.cdisc.org**

CEBS; **http://cebs.niehs.nih.gov**

CTD; **http://ctd.mdibl.org**

DSSTox Coordinating Public Effort project; **http://www.epa.gov/nheerl/dsstox/CoordinatingPublicEfforts.html**

DSSTox; **http://www.epa.gov/nheerl/dsstox**

EBI toxicogenomics; **http://www.ebi.ac.uk/microarray/Projects/tox-nutri**

EBI; **http://www.ebi.ac.uk**

ECVAM; **http://ecvam.jrc.cec.eu.int**

Editorial. 2002. Microarray standards at last. *Nature* **419**: 323.

Editorial. 2003a. EU starts a chemical reaction. *Science* **300**, **5618**: 405.

Editorial. 2003b. Europe whittles down plans for massive chemical testing program. *Science* **302**, **5647**: 969.

Edgar R, Domrachev M, Lash AE. 2002. Gene Expression Omnibus: NCBI gene expression and hybridization array data repository. *Nucleic Acids Res* **30**(1): 207–210.

EPA DRAFT — Potential implications of genomics for regulatory and risk assessment applications at EPA; **http://www.epa.gov/osa/genomics.htm**

ERCC; **http://www.cstl.nist.gov/biotech/workshops/ERCC2004**

FDA draft guidance for industry pharmacogenomic data submissions; **http://www.fda.gov/cder/guidance/5900dft.doc**

Hermjakob H, Montecchi-Palazzi L, Bader G, *et al*. 2004. The HUPO PSI molecular interaction format — a community standard for the representation of protein interaction data. *Nature Biotechnol* **22**: 177–183.

ICCVAM; **http://iccvam.niehs.nih.gov/home.htm**

Ikeo K, Ishi-i J, Tamura T, *et al*. 2003. CIBEX: center for information biology gene expression database. *C R Biol* **326**(10–11): 1079–1082.

ILSI HESI; **http://hesi.ilsi.org/index.cfm?pubentityid=120**

IPCS; **http://www.who.int/ipcs/en**

Kepler; **http://kepler-project.org**

KNB; **http://knb.ecoinformatics.org**

LGC; **http://www.lgc.co.uk**

MARG; **http://www.abrf.org/index.cfm/group.show/Microarray.30.htm**

Copyright © 2005 John Wiley & Sons, Ltd. 

Mattes WB, Pettit SD, Sansone A, *et al*. 2004. Database development in toxicogenomics: issues and efforts. *Environ Health Perspect* **112**: 495–505.

maxd; **http://bioinf.man.ac.uk/microarray/maxd**

MfB; **http://www.mfbprog.org.uk**

MGED RSBI Working Groups; **http://www.mged.org/Workgroups/rsbi**

MGED; **http://www.mged.org**

NAS Committee on Emerging Issues and Data on Environmental Contaminants; **http://dels.nas.edu/emergingissues/index.asp**

NCTR-FDA; **http://www.fda.gov/nctr**

NERC EGTDC; **http://envgen.nox.ac.uk**

NICEATM; **http://iccvam.niehs.nih.gov/home.htm**

NIEHS-NCT; **http://www.niehs.nih.gov/nct**

OECD; **http://www.oecd.org**

Pennie W, Pettit SD, Lord PG. 2004. Toxicogenomics in risk assessment: an overview of an HESI collaborative research program. *Environ Health Perspect* **112**: 417–419.

PSI; **http://psidev.sourceforge.net**

Quackenbush J. 2004. Data standards for 'omic' science. *Nature Biotechnol* **22**: 613–614.

SEEK; **http://seek.ecoinformatics.org**

SEND; **http://www.cdisc.org/models/send/v1.5**

SMRS; **http://www.smrsgroup.org**

SOFG; **http://www.sofg.org**

Spellman PT, Miller M, Stewart J, *et al*. 2002. Design and implementation of microarray gene expression mark-up language (MAGE-ML). *Genome Biol* **3**(9): research0046.

Stoeckert CJ, Parkinson H. 2003. The MGED ontology: a framework for describing functional genomics experiments. *Comp Funct Genom* **4**: 127–132.

Stoeckert CJ, Causton HC, Ball CA. 2002. Microarray databases: standards and ontologies. *Nature Genet* **32**: 469–473.

Tong W, Cao X, Harris S, *et al*. 2003. ArrayTrack — supporting toxicogenomic research at the U.S. Food and Drug Administration National Center for Toxicological Research. *Environ Health Perspect* **111**: 1819–1826.

Tox-MIAMExpress; **http://www.ebi.ac.uk/tox-miamexpress**

Waters M, Boorman G, Bushel P, *et al*. 2003. Systems toxicology and the chemical effects in biological systems knowledge base. *Environ Health Perspect* **111**: 811–824.

Waters MD, Fostel JM. 2004. Toxicogenomics and systems toxicology: aims and prospects. *Nature Rev Genet* **5**: 938–948.

Xirasagar S, Gustafson S, Merrick AB, *et al*. 2004. CEBS object model for systems biology data, CEBS SysBio-OM. *Bioinformatics* **20**(13): 2004–2015.

Copyright © 2005 John Wiley & Sons, Ltd. 

## References

[PDF_00677] Ball Catherine A., Sherlock Gavin, Parkinson Helen, Rocca-Sera Philippe, Brooksbank Catherine, Causton Helen C., Cavalieri Duccio, Gaasterland Terry, Hingamp Pascal, Holstege Frank (2002). Standards for microarray data.. Science.

[PDF_00683] Brazma A., Hingamp P., Quackenbush J., Sherlock G., Spellman P., Stoeckert C., Aach J., Ansorge W., Ball C. A., Causton H. C. (2001). Minimum information about a microarray experiment (MIAME)-toward standards for microarray data.. Nat Genet.

[PDF_00680] Brazma Alvis, Parkinson Helen, Sarkans Ugis, Shojatalab Mohammadreza, Vilo Jaak, Abeygunawardena Niran, Holloway Ele, Kapushesky Misha, Kemmeren Patrick, Lara Gonzalo Garcia (2003). ArrayExpress--a public repository for microarray gene expression data at the EBI.. Nucleic Acids Res.

[PDF_00702] Edgar Ron, Domrachev Michael, Lash Alex E. (2002). Gene Expression Omnibus: NCBI gene expression and hybridization array data repository.. Nucleic Acids Res.

[PDF_00711] Hermjakob Henning, Montecchi-Palazzi Luisa, Bader Gary, Wojcik Jérôme, Salwinski Lukasz, Ceol Arnaud, Moore Susan, Orchard Sandra, Sarkans Ugis, von Mering Christian (2004). The HUPO PSI's molecular interaction format--a community standard for the representation of protein interaction data.. Nat Biotechnol.

[PDF_00716] Ikeo Kazuho, Ishi-i Jun, Tamura Takurou, Gojobori Takashi, Tateno Yoshio (2003). CIBEX: center for information biology gene expression database.. C R Biol.

[PDF_00728] Mattes William B., Pettit Syril D., Sansone Susanna-Assunta, Bushel Pierre R., Waters Michael D. (2004). Database development in toxicogenomics: issues and efforts.. Environ Health Perspect.

[PDF_00743] Pennie William, Pettit Syril D., Lord Peter G. (2004). Toxicogenomics in risk assessment: an overview of an HESI collaborative research program.. Environ Health Perspect.

[PDF_00747] Quackenbush John (2004). Data standards for 'omic' science.. Nat Biotechnol.

[PDF_00753] Spellman Paul T., Miller Michael, Stewart Jason, Troup Charles, Sarkans Ugis, Chervitz Steve, Bernhart Derek, Sherlock Gavin, Ball Catherine, Lepage Marc (2002). Design and implementation of microarray gene expression markup language (MAGE-ML).. Genome Biol.

[PDF_00759] Stoeckert Christian J., Causton Helen C., Ball Catherine A. (2002). Microarray databases: standards and ontologies.. Nat Genet.

[PDF_00756] Stoeckert Christian J., Parkinson Helen (2003). The MGED ontology: a framework for describing functional genomics experiments.. Comp Funct Genomics.

[PDF_00761] Tong Weida, Cao Xiaoxi, Harris Stephen, Sun Hongmei, Fang Hong, Fuscoe James, Harris Angela, Hong Huixiao, Xie Qian, Perkins Roger (2003). ArrayTrack--supporting toxicogenomic research at the U.S. Food and Drug Administration National Center for Toxicological Research.. Environ Health Perspect.

[PDF_00769] Waters Michael D., Fostel Jennifer M. (2004). Toxicogenomics and systems toxicology: aims and prospects.. Nat Rev Genet.

[PDF_00771] Xirasagar Sandhya, Gustafson Scott, Merrick B. Alex, Tomer Kenneth B., Stasiewicz Stanley, Chan Denny D., Yost Kenneth J., Yates John R., Sumner Susan, Xiao Nianqing (2004). CEBS object model for systems biology data, SysBio-OM.. Bioinformatics.

